# Supplementary material for: Global, regional, and national burden of kidney dysfunction-attributed ischemic heart disease from 1990 to 2021: A systematic analysis of the Global Burden of Disease study 2021
Source: PLoS One. 2025 Dec 29;20(12):e0335548. doi: 10.1371/journal.pone.0335548 (PMC12747353; doi:10.1371/journal.pone.0335548)
Supplement: Attachment — Submitted filename: Response_to_Reviewers.docx [file pone.0335548.s004.docx]

**Title:** Global, regional, and national burden of kidney dysfunction-attributed ischemic heart disease from 1990 to 2021: a systematic analysis of the Global Burden of Disease study 2021

**ID:** PONE-D-25-23351

Dear Editor,

Thanks for providing us with this great opportunity to submit a revised version of our manuscript. We appreciate the detailed and constructive comments provided by the reviewers. We have carefully revised the manuscript by incorporating all the suggestions by the review panel.

In response to the comments, we have conducted a thorough literature review and detailed discussion, and revised the manuscript accordingly, with the changes highlighted in red.

We hope that our responses have adequately addressed the reviewers’ concerns and look forward to publishing our manuscript in your esteemed journal. Please do not hesitate to contact me if you require further clarification on any of the above items or have additional questions.

Kind regards,

Ying Zhang, M.D.

Department of Cardiology, Heze Municipal Hospital, Heze 274031, Shandong, China

Email: [zhyinglove@163.com](mailto:zhyinglove@163.com)

**Editorial Requirements – Formatting and Data Sharing**

*1. Please ensure that your manuscript meets PLOS ONE's style requirements, including those for file naming*

**Response：**We standardized the format according to the templates provided in the information for authors. We have carefully revised the manuscript to ensure full compliance with the PLOS ONE formatting guidelines. This includes restructuring the manuscript sections, standardizing figure legends, and ensuring appropriate file naming conventions (e.g., Main_manuscript.docx, Supporting_information.docx, and separate high-resolution figure files). The title and author affiliations are also formatted according to the official template provided by PLOS ONE.

*2. Thank you for uploading your study's underlying data set. Unfortunately, the repository you have noted in your Data Availability statement does not qualify as an acceptable data repository according to PLOS's standards. At this time, please upload the minimal data set necessary to replicate your study's findings to a stable, public repository (such as figshare or Dryad) and provide us with the relevant URLs, DOIs, or accession numbers that may be used to access these data*

**Response：**We appreciate your guidance regarding data deposition. All data used in this study are publicly available and freely accessible through the Global Burden of Disease (GBD) Results Tool provided by the Institute for Health Metrics and Evaluation (IHME). We have revised our data availability statement based on the latest publication from PLOS One (lines 405-409).

Specifically, the minimal dataset used to reproduce our analyses—including age-standardized DALYs, mortality rates, and population estimates attributable to kidney dysfunction—can be accessed at: <http://ghdx.healthdata.org/gbd-results-tool>. No proprietary or restricted data were used. The revised Data Availability Statement in the manuscript has been updated to reflect this (lines 405-409). If it still does not meet PLOS standards, please contact us to resolve the issue.

**Response to Reviewers**

**Reviewer #1:**

**Comment 1: *Lines 48–76 (introduction) Condense background on IHD to 2–3 concise lines and focus on the novelty of KD as an emerging factor.***

**Response:** Thank you for this suggestion. We have condensed the background on ischemic heart disease (IHD) to three concise lines, restructured the Introduction to emphasize kidney dysfunction (KD) as an emerging and independent risk factor, and highlighted the novelty of assessing KD-attributable IHD from a global and SDI-stratified perspective (lines 47-56).

**Comment 2:** ***Pages 8–15 (DALY/ASR breakdown by region) → Summarize key findings for low, middle, and high SDI groups. Include comparative statements like, “High-SDI countries saw the steepest ASR decline, while low-middle SDI groups showed slight increases.***

**Response:** Thank you for this insightful suggestion. In response, we have added a new comparative summary paragraph to the Results section (lines 225–234), explicitly highlighting the differences in ASDR and ASMR trends across high-, middle-, and low-middle SDI regions.

Specifically, we report that high-SDI countries experienced the steepest declines in age-standardized rates (ASRs)—exceeding 60% reductions between 1990 and 2021—accompanied by the most negative EAPCs. In contrast, low-middle SDI regions showed slight but statistically significant increases in both ASDR and ASMR, with small positive EAPCs, suggesting worsening burden. Middle-SDI countries demonstrated relatively stable trends with minimal changes in ASRs.

**Comment 3:** ***While the Discussion section recognizes disparities, the policy-level implications are generic (e.g., "integrated cardiovascular and renal care") without specifying how they may be implemented. Suggestion: Add region-specific recommendations (e.g., for sub-Saharan Africa: community-level hypertension screening programs linked with CKD testing). Consider citing existing successful models (e.g., WHO HEARTS initiative) or specific public health programs. Lines 322–334 → Expand on practical intervention models suitable for different SDI regions.***

**Response:** Thank you for this thoughtful and constructive suggestion. In response, we have substantially expanded the Discussion section (lines 327–368) to provide region-specific recommendations aligned with differing SDI contexts.

For low-SDI regions such as sub-Saharan Africa, we now recommend implementing community-level hypertension and diabetes screening programs integrated with CKD testing at the primary care level, supported by mobile clinics and task-shifting to non-physician health workers. We cite the WHO HEARTS initiative as a proven, scalable framework for integrating cardiovascular and renal care in such settings [1-3].

In middle-SDI regions, including South Asia and Latin America, we highlight the India Hypertension Control Initiative (IHCI) and Sri Lanka’s national NCD platform as effective models for embedding CKD management into broader noncommunicable disease care using protocol-based treatment and digital tools. These approaches are discussed as examples for scale-up [4-5].

In high-SDI countries, we recommend integrating CKD care into existing cardiovascular pathways, enhancing electronic health record (EHR)–based risk stratification, and addressing residual disparities through sex- and age-specific interventions [6-8]. We also emphasize incorporating SGLT2 inhibitors into standard protocols, given their proven reno- and cardioprotective effects [9].

These additions are intended to enhance the policy relevance and translational value of our findings by demonstrating how CKD-attributable IHD prevention strategies can be operationalized within diverse health system contexts.

***References***

[1]. Wellmann IA, Ayala LF, Valley TM, et al. Evaluating the World Health Organization's Hearts Model for Hypertension and Diabetes Management: A Pilot Implementation Study in Guatemala. Glob Heart. 2025;20(1):9. Published 2025 Jan 31. doi:10.5334/gh.1397.

[2]. Devi R, Kanitkar K, Narendhar R, Sehmi K, Subramaniam K. A Narrative Review of the Patient Journey Through the Lens of Non-communicable Diseases in Low- and Middle-Income Countries. Adv Ther. 2020;37(12):4808-4830. doi:10.1007/s12325-020-01519-3.

[3]. Al-Ghamdi S, Abu-Alfa A, Alotaibi T, et al. Chronic Kidney Disease Management in the Middle East and Africa: Concerns, Challenges, and Novel Approaches. Int J Nephrol Renovasc Dis. 2023;16:103-112. doi:10.2147/IJNRD.S363133.

[4]. Mohan V, Seedat YK, Pradeepa R. The rising burden of diabetes and hypertension in southeast asian and african regions: need for effective strategies for prevention and control in primary health care settings. Int J Hypertens. 2013;2013:409083. doi:10.1155/2013/409083.

[5]. Ameh OI, Ekrikpo UE, Kengne AP. Preventing CKD in Low- and Middle-Income Countries: A Call for Urgent Action. Kidney Int Rep. 2019;5(3):255-262. doi:10.1016/j.ekir.2019.12.013.

[6]. Narva AS, Briggs M. The National Kidney Disease Education Program: improving understanding, detection, and management of CKD. Am J Kidney Dis. 2009;53(3 Suppl 3):S115-S120. doi:10.1053/j.ajkd.2008.05.038.

[7]. German CA, Baum SJ, Ferdinand KC, et al. Defining preventive cardiology: A clinical practice statement from the American Society for Preventive Cardiology. *Am J Prev Cardiol*. 2022;12:100432. doi:10.1016/j.ajpc.2022.100432.

[8]. Zheng X, Yang Z, Liu S, Li Y, Wang A. Digital symptom management interventions for people with chronic kidney disease: a scoping review based on the UK Medical Research Council Framework. *BMC Public Health*. 2024;24(1):3534. doi:10.1186/s12889-024-20871-5.

[9]. Badve SV, Bilal A, Lee MMY, et al. Effects of GLP-1 receptor agonists on kidney and cardiovascular disease outcomes: a meta-analysis of randomised controlled trials. Lancet Diabetes Endocrinol. 2025;13(1):15-28. doi:10.1016/S2213-8587(24)00271-7.

**Comment 4:** ***Switch consistently between “KD-related IHD” or “KD-attributable IHD.” Both are used interchangeably—standardize.***

**Response:** Thank you for pointing out this important issue. To ensure epidemiological consistency and clarity, we have standardized the terminology throughout the manuscript to “KD-attributable IHD”. This term aligns with the Global Burden of Disease (GBD) framework, which quantifies disease burden based on attributable risk estimation models. Using “attributable” more accurately reflects causal contributions from kidney dysfunction to IHD, as estimated by comparative risk assessments in the GBD methodology.

**Comment 5:** ***Include short summary captions for each figure (e.g., “Trends in DALYs by SDI group”).***

**Response:** Thank you for this helpful suggestion. In accordance with your recommendation, we have revised the figure legends throughout the Results section to include concise and descriptive summary captions.

Each updated figure legend now begins with a brief overview sentence that summarizes the main message of the figure, followed by concise panel descriptions where applicable. We also ensured consistent definitions and expansions of abbreviations such as ASDR, ASMR, SDI, and DALYs, to improve clarity and reader accessibility.

**Reviewer #2** This manuscript provides a comprehensive analysis of the epidemiological trends and global disparities in the burden of ischemic heart disease (IHD) attributable to kidney dysfunction (KD). The study is timely and relevant, addressing a neglected area in cardiovascular-renal epidemiology. But, there are certain.

**Comment 1: *Consideration of key subpopulations such as individuals with diabetes or hypertension—known shared risk factors for CKD and IHD—could further enrich the analysis.***

**Response:** Thank you for your valuable comment. We fully agree that hypertension and diabetes are important comorbidities that contribute significantly to both chronic kidney disease (CKD) and ischemic heart disease (IHD). These conditions share common pathophysiological mechanisms—such as endothelial dysfunction, systemic inflammation, and metabolic dysregulation—which may synergistically amplify cardiovascular risk, especially in populations with multiple chronic conditions.

As the reviewer rightly points out, the comparative risk assessment (CRA) framework used in the Global Burden of Disease (GBD) study estimates the burden attributable to each risk factor independently. Although this approach incorporates adjustments for confounders and comorbidities, it does not capture interaction or synergistic effects between overlapping exposures, such as concurrent kidney dysfunction and hypertension or diabetes [1, 2]. This may lead to an underestimation of the true IHD burden in multimorbid individuals.

In response to this important observation, we have made the following revisions to the manuscript:

In the Introduction, we now highlight that, unlike well-characterized metabolic risk factors such as elevated systolic blood pressure and high fasting glucose [3, 4], kidney dysfunction remains under-assessed in the context of multimorbidity (lines 50-52);

In the Limitations section, we explicitly acknowledge that the GBD framework does not account for complex interactions among risk factors, and we recommend future studies to apply joint risk modeling or mediation analysis to better understand these relationships and improve causal attribution (lines 382-389).

***References***

[1] GBD 2021 Risk Factors Collaborators. Global burden and strength of evidence for 88 risk factors in 204 countries and 811 subnational locations, 1990-2021: a systematic analysis for the Global Burden of Disease Study 2021. Lancet. 2024;403(10440):2162-2203. doi:10.1016/S0140-6736(24)00933-4.

[2] Safiri S, Karamzad N, Singh K, et al. Burden of ischemic heart disease and its attributable risk factors in 204 countries and territories, 1990-2019. Eur J Prev Cardiol. 2022;29(2):420-431. doi:10.1093/eurjpc/zwab213.

[3] Zhuang Z, Wang Q, Li H, Lan S, Su Y, Lin Y, Guo P: Global trends and disparities in ischemic heart disease attributable to high systolic blood pressure, 1990-2021: Insights from the global burden of disease study. PLoS One 2025, 20(6):e0324073.

[4] Wang L, Ma N, Wei L: Global burden of ischemic heart disease attributable to high sugar-sweetened beverages intake from 1990 to 2019. Nutr Metab Cardiovasc Dis 2023, 33(6):1190-1196.

**Comment 2:** ***The authors could have added a brief discussion elaborating on pathophysiological mechanisms linking KD and IHD, and reviewing the evidence for specific interventions that may reduce the attributable burden (e.g., ACE inhibitors, SGLT2 inhibitors).***

**Response:** Thank you for this valuable suggestion. In response, we have expanded the Discussion section (lines 252-262) to summarize the principal pathophysiological mechanisms by which KD increases cardiovascular risk. These include endothelial dysfunction, chronic systemic inflammation, oxidative stress, arterial stiffness, and calcium-phosphate imbalance—all of which promote vascular calcification and accelerated coronary atherosclerosis, contributing to impaired myocardial perfusion and increased IHD burden [1,2].

Furthermore, we have included a concise summary of recent clinical evidence supporting the use of renin–angiotensin–aldosterone system (RAAS) inhibitors and sodium-glucose cotransporter 2 (SGLT2) inhibitors. These agents have been shown to reduce cardiovascular events, delay CKD progression, and lower all-cause mortality in high-risk populations, including those without diabetes [3]. Based on this evidence, we recommend their routine integration into cardiorenal treatment protocols, particularly in high-SDI settings where access to these medications is more feasible (lines 352-356).

***References***

[1]. Jankowski J, Floege J, Fliser D, Böhm M, Marx N: Cardiovascular Disease in Chronic Kidney Disease: Pathophysiological Insights and Therapeutic Options. Circulation 2021, 143(11):1157-1172.

[2]. Zoccali C, Mallamaci F, Adamczak M, de Oliveira RB, Massy ZA, Sarafidis P, Agarwal R, Mark PB, Kotanko P, Ferro CJ et al: Cardiovascular complications in chronic kidney disease: a review from the European Renal and Cardiovascular Medicine Working Group of the European Renal Association. Cardiovasc Res 2023, 119(11):2017-2032.

[3]. Badve SV, Bilal A, Lee MMY, et al. Effects of GLP-1 receptor agonists on kidney and cardiovascular disease outcomes: a meta-analysis of randomised controlled trials. Lancet Diabetes Endocrinol. 2025;13(1):15-28. doi:10.1016/S2213-8587(24)00271-7.

**Comment 3: *While the GBD framework offers standardized and widely accepted estimates, it is important to recognize that data quality and availability can vary substantially across regions, particularly in low- and middle-income countries. The authors could have acknowledged this limitation and consider the potential for estimation bias in these settings where health surveillance systems may be less robust.***

**Response:** This is an important point. While the GBD framework offers a standardized and internally consistent approach to estimating disease burden across regions, the accuracy and precision of these estimates are inherently influenced by the quality, availability, and representativeness of primary data sources, which can vary considerably across geographies.

The GBD study applies advanced statistical modeling tools—such as DisMod-MR for non-fatal outcomes and the Cause of Death Ensemble model (CODEm) for mortality estimates—to compensate for data gaps and improve internal coherence. These models utilize covariates and borrow strength across time and space to generate plausible estimates even in data-sparse or underreported regions [1]. Nevertheless, in many low- and middle-income countries, where health surveillance systems may be weak or incomplete, these model-based estimates are subject to greater uncertainty and potential bias.

In the revised Discussion section, we now explicitly acknowledge this limitation and emphasize that interpretations of subnational or regional differences should be made cautiously, given the potential for estimation bias. We also note that improvements in health data infrastructure and routine surveillance—particularly in low- and middle-income countries—remain essential for improving the reliability of future burden assessments (lines 371-376).

***References***

[1] GBD 2021 Risk Factors Collaborators. Global burden and strength of evidence for 88 risk factors in 204 countries and 811 subnational locations, 1990-2021: a systematic analysis for the Global Burden of Disease Study 2021. Lancet. 2024;403(10440):2162-2203. doi:10.1016/S0140-6736(24)00933-4.

**Comment 4:** ***The authors could have added a brief discussion on the strategies followed in the high income countries in for identifying KD.***

**Response:** We agree that early detection strategies for kidney dysfunction (KD) in high-income countries (HICs) are essential for reducing the downstream burden of KD-attributable ischemic heart disease (IHD). In the revised Discussion section (lines 347-365), we have added a detailed paragraph outlining policy-level strategies and clinical interventions that are widely adopted in high-SDI settings.

Specifically, we discuss the integration of CKD screening into existing cardiovascular disease management pathways, ensuring continuity of care from primary screening to specialist follow-up. We also highlight targeted measures aimed at addressing residual disparities, such as age- and sex-specific education campaigns and the use of electronic health record (EHR) systems to trigger risk-based screening reminders [1-3].

In addition, we emphasize the importance of incorporating sodium-glucose cotransporter-2 inhibitors (SGLT2is) into standard treatment protocols, given their proven cardiorenal benefits [4]. Furthermore, we recommend embedding KD prevention strategies within broader non-communicable disease (NCD) platforms, supported by multisectoral policies—including salt reformulation, sugar taxes, tobacco control, and improved access to essential medicines such as antihypertensives and statins.

***References***

[1]. Narva AS, Briggs M. The National Kidney Disease Education Program: improving understanding, detection, and management of CKD. Am J Kidney Dis. 2009;53(3 Suppl 3):S115-S120. doi:10.1053/j.ajkd.2008.05.038.

[2]. German CA, Baum SJ, Ferdinand KC, et al. Defining preventive cardiology: A clinical practice statement from the American Society for Preventive Cardiology. Am J Prev Cardiol. 2022;12:100432. doi:10.1016/j.ajpc.2022.100432.

[3]. Zheng X, Yang Z, Liu S, Li Y, Wang A. Digital symptom management interventions for people with chronic kidney disease: a scoping review based on the UK Medical Research Council Framework. BMC Public Health. 2024;24(1):3534. doi:10.1186/s12889-024-20871-5.

[4]. Badve SV, Bilal A, Lee MMY, et al. Effects of GLP-1 receptor agonists on kidney and cardiovascular disease outcomes: a meta-analysis of randomised controlled trials. Lancet Diabetes Endocrinol. 2025;13(1):15-28. doi:10.1016/S2213-8587(24)00271-7.

**Comment 5: *A brief discussion on the policy integrated cardiovascular and renal care, particularly in resource-limited settings, which aligns with global health priorities could be better.***

**Response:** Thank you for highlighting this important point. To address this, we have further expanded the Discussion section (Lines 329-369) to explicitly discuss how integrated cardiovascular and renal care models—particularly in resource-limited settings—can be aligned with global health priorities, such as those outlined in the WHO Global Action Plan for the Prevention and Control of Noncommunicable Diseases.

We now describe practical examples of integrated care platforms that have been implemented or scaled in low- and middle-income settings. These include the WHO HEARTS initiative, which provides a standardized package for CVD prevention and has been adapted to include kidney health interventions in primary care contexts. In addition, we reference the India Hypertension Control Initiative (IHCI) and Sri Lanka’s national NCD program as successful models that embed chronic kidney disease (CKD) screening and management within broader NCD frameworks using protocol-based care and task-sharing strategies.

Importantly, we emphasize that such integrated approaches offer dual cardiovascular and renal benefits, and are particularly effective when tailored to local epidemiologic trends and health system capacity. These enhancements ensure that the policy recommendations presented in our study are both globally relevant and contextually adaptable, thereby reinforcing their alignment with ongoing global health initiatives.
