# Supplementary material for: Global, regional, and national burden of kidney dysfunction-attributed ischemic heart disease from 1990 to 2021: A systematic analysis of the Global Burden of Disease study 2021
Source: PLoS One. 2025 Dec 29;20(12):e0335548. doi: 10.1371/journal.pone.0335548 (PMC12747353; doi:10.1371/journal.pone.0335548)
Supplement: Attachment — Submitted filename: Response_to_Reviewers_auresp_2.docx [file pone.0335548.s005.docx]

**Title:** Global, regional, and national burden of kidney dysfunction-attributed ischemic heart disease from 1990 to 2021: a systematic analysis of the Global Burden of Disease study 2021

**ID:** PONE-D-25-23351R1

Dear Editor,

Thanks for providing us with this great opportunity to submit a revised version of our manuscript. We appreciate the detailed and constructive comments provided by the reviewers. We have carefully revised the manuscript by incorporating all the suggestions by the review panel.

In response to the comments, we have conducted a thorough literature review and detailed discussion, and revised the manuscript accordingly, with the changes highlighted in red.

We hope that our responses have adequately addressed your concerns and look forward to publishing our manuscript in your esteemed journal. Please do not hesitate to contact me if you require further clarification on any of the above items or have additional questions.

Kind regards,

Ying Zhang, M.D.

Department of Cardiology, Heze Municipal Hospital, Heze 274031, Shandong, China

Email: zhyinglove@163.com

**Journal Requirements:**

If the reviewer comments include a recommendation to cite specific previously published works, please review and evaluate these publications to determine whether they are relevant and should be cited. There is no requirement to cite these works unless the editor has indicated otherwise.

The reviewers' comments did not include suggestions for citing specific previously published works.

*Please review your reference list to ensure that it is complete and correct. If you have cited papers that have been retracted, please include the rationale for doing so in the manuscript text, or remove these references and replace them with relevant current references. Any changes to the reference list should be mentioned in the rebuttal letter that accompanies your revised manuscript. If you need to cite a retracted article, indicate the article’s retracted status in the References list and also include a citation and full reference for the retraction notice.*

We have checked the reference list to ensure it is complete and accurate. We have not cited any retracted papers.

**Editor Comments**

*1. Introduction: To improve the context, I suggest the authors using/updating this section with recent studies which have emphasized the association of CKD in both moderate and severe stages with adverse cardiovascular events (DOI: 10.1161/JAHA.125.042307 ; DOI: 10.34067/KID.0000000705 )*

**Response：**Thank you for sharing. To improve the context, we revised the introduction by referring to these two papers (DOI: 10.1161/JAHA.125.042307; DOI: 10.34067/KID.0000000705) (**lines57-63**).

*2. Methodology:*

*2.1 A brief discussion about data collecting protocol specifying which keywords and how did you collect the data might be useful for readers and other scientists to replicate similar studies in the future.*

**Response：**Thank you for your suggestion. We have added this clarification to the Methods section (**lines 81-85**).

*2.2 Please also specify the formula of your parameters such as ASR, EAPC. Moreover, I believe most of data from GBD provide you 95% of Uncertainty Interval which is quite different than Confidence Interval. Please also explain this estimate.*

**Response：**Thank you for your valuable feedback. As requested, we would like to clarify the details regarding the Age-Standardized Rate (ASR) calculation. The ASR was provided directly from the Global Health Data Exchange (GHDx), as part of the data from the Global Burden of Disease (GBD) Study 2021. We did not perform the calculation of ASR ourselves, but rather obtained the pre-calculated rates from the GBD database, which uses the method outlined in their official documentation. The ASR formula provided by GHDx is:

The EAPC calculation formula has been supplemented in the statistical analysis section (**lines 136-140**).

You are correct that GBD data provides Uncertainty Intervals (UI) instead of Confidence Intervals (CI), and we acknowledge the difference. All GBD data, including the absolute numbers, rates, and ASRs of DALYs and deaths, provide 95% UI. Our calculations based on the data provided by GBD include 95% CI. We have supplemented the explanation of the 95% UI in the descriptive analysis section (**lines 115-123**).

Uncertainty Intervals (UI) reflect the range within which the true value is expected to lie, incorporating not only statistical variability (such as sampling error) but also the systematic uncertainties in the model assumptions, data sources, and input variables. The GBD study utilizes a probabilistic model to estimate health outcomes, and the UI accounts for all potential sources of uncertainty, including variations in the input data, modeling techniques, and the assumptions made during the estimation process. The 95% UI thus represents a range of possible values for a given health estimate, with 95% of possible estimates expected to fall within this interval.

Confidence Intervals (CI), by contrast, are generally derived from statistical sampling theory and primarily reflect the uncertainty due to random sampling error. A 95% CI indicates that if the same study were repeated multiple times, the true value would fall within the interval 95% of the time. However, CI does not account for the systematic uncertainties in the data or the modeling process.

Given the complexity of global health data, which integrates multiple sources and modeling approaches, Uncertainty Intervals offer a more comprehensive understanding of the variability in our estimates. By including both statistical and model-related uncertainties, 95% UIs provide a more realistic range of possible outcomes, particularly for complex epidemiological estimates like those derived from the GBD study.

*3 Results/Discussion*

*3.1 The observed sex differences are interesting and consistent with previous studies. Existing evidence indicates that the prevalence of CKD is higher in females than in males, likely due to longer life expectancy and potential overdiagnosis (DOI: 10.1038/nrneph.2017.181). However, males carry a substantially greater risk of adverse cardiovascular events. Your findings align well with this established knowledge. I recommend expanding the discussion (lines 261–271) to highlight the higher cardiovascular risk in males and provide additional rationale for this observation. This represents one of the major findings of your study and warrants more in-depth discussion.*

**Response：**Thank you for your insightful feedback and suggestions. In response to your recommendation, we have expanded the discussion in **lines 282–303** to highlight the higher cardiovascular risk observed in males despite the higher prevalence of chronic kidney disease (CKD) in females. We have provided additional rationale to explain this finding, based on several key factors that contribute to the observed disparity:

1. Differences in CKD Prevalence: We acknowledge that women have a higher prevalence of CKD, which is partly influenced by demographic factors such as longer life expectancy and the overestimation of CKD in low-muscle-mass individuals due to creatinine-based eGFR equations.

2. Faster Progression in Men: Men tend to progress more rapidly to advanced CKD and are more likely to accumulate cardiometabolic comorbidities, such as poorly controlled hypertension, diabetes, and smoking. These factors significantly increase their risk of ischemic events and premature cardiovascular death.

3. Behavioral and Care-Seeking Differences: Men also tend to present later for medical care and are less likely to adhere to preventive therapies, which further exacerbates their cardiovascular risk.

4. Biological Differences: We expanded on the biological mechanisms at play, including the vasculoprotective effects of estrogen in women versus the potentially harmful effects of androgens, as well as sex differences in endothelial function, oxidative stress, and immune responses.

5. Health-System and Selection Effects: Additionally, we discussed how differential referral patterns and the timing of renal-replacement therapy can influence outcomes, with the male disadvantage becoming more pronounced as patients age.

We believe that these expanded points better support the observed findings and provide a more comprehensive understanding of the higher cardiovascular risk in males with kidney dysfunction. We have incorporated these ideas to further emphasize the need for sex-sensitive strategies in managing kidney disease and ischemic heart disease, especially in high-risk men.

Thank you again for your valuable input. We trust that these revisions strengthen the manuscript and address your concerns.
